# The influence of folate pathway polymorphisms on high-dose methotrexate-related toxicity and survival in children with non-Hodgkin malignant lymphoma

**DOI:** 10.2478/raon-2013-0076

**Published:** 2014-07-10

**Authors:** Nina Erculj, Barbara Faganel Kotnik, Marusa Debeljak, Janez Jazbec, Vita Dolzan

**Affiliations:** 1 Pharmacogenetics Laboratory, Institute of Biochemistry, Faculty of Medicine, University of Ljubljana, Ljubljana, Slovenia; 2 Department of Hematology and Oncology, University Children’s Hospital, University Medical Centre, Ljubljana, Slovenia; 3 Centre for Medical Genetics, University Children’s Hospital, University Medical Centre, Ljubljana, Slovenia

**Keywords:** childhood, non-Hodgkin lymphoma, folate pathway, polymorphism, high-dose methotrexate, toxicity

## Abstract

**Background:**

We evaluated the influence of folate pathway polymorphisms on high-dose methotrexate (HD-MTX) related toxicity in paediatric patients with T-cell non-Hodgkin lymphoma (NHL).

**Patients and methods:**

In total, 30 NHL patients were genotyped for selected folate pathway polymorphisms.

**Results:**

Carriers of at least one *MTHFR* 677T allele had significantly higher MTX area under the time-concentration curve levels at third MTX cycle (*P* = 0.003). These patients were also at higher odds of leucopoenia (*P* = 0.006) or thrombocytopenia (*P* = 0.041) and had higher number of different HD-MTX-related toxicity (*P* = 0.035) compared to patients with wild-type genotype.

**Conclusions:**

Our results suggest an important role of *MTHFR* 677C>T polymorphism in the development of HD-MTX-related toxicity in children with NHL.

## Introduction

Lymphomas are the second most common group of cancers in children and adolescents in Slovenia (www.slora.si). Non-Hodgkin’s lymphomas (NHLs) represent approximately 50% of these diagnoses in children and have an average 10-year incidence in Slovenia of six cases per year. NHL comprises a heterogeneous group of lymphoid neoplasms.^1^ Among the three major subgroups of childhood NHL according to the World Health Organization Classification, children with lymphoblastic lymphoma of the precursor B- or T-cell types (LBL) are treated according to childhood acute lymphoblastic leukaemia (ALL) protocols^2^, which include administration of high-dose methotrexate (HD-MTX).

MTX is folate analogue, which inhibits several enzymes of the folate pathway. The imbalance of folate homeostasis may lead to DNA synthesis arrest and could disrupt DNA and protein methylation reaction. Our previous studies showed that genetic variability in the key folate pathway enzymes, 5,10-methylenetetrahydrofolate dehydrogenase 1 (MTHFD1), 5,10-methylenetetrahydrofolate reductase (MTHFR), and thymidylate synthase (TYMS), might influence MTX pharmacokinetics and treatment outcome in children with ALL.^3^,^4^ Therefore, the aim of the present study was to assess the influence of polymorphisms in these genes on HDMTX-related toxicity in children with NHL treated according to Berlin-Frankfurt-Münster (BFM) protocols.

## Patients and methods

The cohort consisted of all the patients with primary NHL aged ≤ 18 years at the time of diagnosis who were diagnosed and treated between the years 1993 and 2009 at the Department of Hematology and Oncology, University Children‘s Hospital, Ljubljana, Slovenia. Only patients with lymphoblastic T-cell NHL treated according to the BFM protocols^3^ who received HD-MTX during consolidation phase were included into the study group. Toxicity evaluation and HD-MTX pharmacokinetics data collection and analysis were described previously.^4^ All the subjects and/or their parents or legal guardians gave their written informed consent to participate in the study. The study was approved by the Slovenian Ethics Committee for Research in Medicine and was carried out according to the Declaration of Helsinki.

Genomic DNA was isolated from archived bone marrow slides using QIAamp DNA Mini kit (Qiagen).^5^ *MTHFD1* 1958G>A (rs2236225), *MTHFR* 677C>T (rs1801133), and 1298A>C (rs1801131) were determined by TaqMan SNP genotyping method (Applied Biosystems, Foster City, CA, USA). *TYMS* 2R>3R (rs34743033) and 3RG>3RC (rs2853542) polymorphisms were genotyped as previously described.^4^

For each polymorphism, deviation of genotype frequency distribution from those expected under Hardy-Weinberg equilibrium (HWE) was assessed using standard chi-square test. To test the difference between two groups the Student’s t-test was used in case of normally distributed data and the nonparametric correlations were used for non-normally distributed data. Associations of investigated polymorphisms with HD-MTX-related toxicity were examined using logistic regression models.^4^ The level of significance was set to 0.050. All statistical analyses were carried out by SPSS for Windows, version 19.0 (Statistical Package for the Social Sciences, Chicago, IL).

## Results

### Patients

Among the entire cohort of 76 children with NHL, 29 patients with lymphoblastic T-cell NHL were included into study group as they were treated according to BFM protocols. For one patient, no treatment-related data were available. In total, patients received 110 cycles of HD-MTX. Clinical and treatment characteristics of the study group are summarized in Table 1.

HD-MTX-related toxicity was observed in 25 (86.2%) patients and the number of different toxicities in individual patients ranged from one to five. Prevalence of HD-MTX-related toxicities is shown in Table 2. Frequencies of all investigated polymorphisms were in HWE and were consistent with published data for Slovenian healthy young individuals^6^ and paediatric patients with ALL.^4^

There were no influences of investigated polymorphisms on Cmax; however, the mean AUC levels at third HD-MTX cycle were significantly higher in carriers of at least one *MTHFR* 677T allele compared to patients with wild-type genotype (mean AUC3 ± standard deviation: 163.7 ± 108.0 μmol*h/l versus 431.7 ± 186.1 μmol*h/l, *P* = 0.003). AUC levels also correlated with the number of different HD-MTX-related toxicities in individual patients (AUC1: τ = 0.423, *P* = 0.015 and AUC3: τ = 0.586, *P* = 0.001). The influence of investigated polymorphisms on HD-MTX-related toxicity is presented in Table 3. *MTHFD1* 1958G>A, *MTHFR* 1298A>C, and *TYMS* polymorphisms were not associated with any of investigated toxicities. In contrast, carriers of *MTHFR* 677T allele had significantly increased odds of leucopoenia grade ≥ 2 (OR = 1.86; 95% CI = 1.12–3.07; *P* = 0.006) and thrombocytopenia grade ≥ 2 (OR = 11.14; 95% CI = 1.11–112.01; *P* = 0.041). The association of thrombocytopenia grade ≥ 2 (OR = 39.42; 95% CI = 2.11–734.94; *P* = 0.014) remained significant in multivariable model adjusted for patients’ age. In addition, the number of polymorphic *MTHFR* 677T alleles positively correlated with number of different HD-MTX-related toxicities in individual patients (τ = 0.442, *P* = 0.010). We did not observe any associations between *MTHFR* haplotypes and HD-MTX-related toxicity (data not shown).

## Discussion

The present study investigated the role of several folate pathway polymorphisms in development of HD-MTX-related toxicity and survival in a group of paediatric patients with lymphoblastic T-cell NHL treated according to BFM protocols.

We did not observe any association of *MTHFD1* or *TYMS* polymorphisms with HD-MTX-related toxicity. In contrast to these results, our previous study showed an influence of *TYMS* 2R>3R polymorphism on different haematotoxicitiesa, as well as an influence of *MTHFD1* 1958G>A polymorphism on hepatotoxicity grade ≥ 2 in paediatric patients with ALL.^4^ The association between *MTHFD1* 1958G>A polymorphism and hepatotoxicity grade ≥ 2 could not be assessed in the present study due to the low frequency of hepatotoxicity grade ≥ 2. Moreover, the lack of association between *TYMS* polymorphisms and treatment outcomes in our study might be due to the low frequency of wild-type 2R/2R genotype and a small study population, leading to insufficient statistical power to detect significant associations.

In concordance with our previous studies, investigating HD-MTX pharmacokinetics^3^ and HDMTX-related toxicity in children with ALL^4^, we observed significantly higher AUC levels in carriers of at least one *MTHFR* 677T allele compared to patients with wild-type genotype. Similar to our findings, other studies also reported the influence of *MTHFR* 677T allele on higher MTX plasma levels.^7^,^8^ In addition, we observed significantly increased odds of leucopoenia and thrombocytopenia, as well as higher number of different HD-MTX-related toxicities in carriers of at least one *MTHFR* 677T allele compared to patients with wild-type geno-type. Although these findings do not support our results obtained in a group of paediatric patients with ALL^4^, it must be noted that compared to ALL patients, patients with NHL were predominantly male, older at the time of diagnosis, and received higher doses of MTX. A few studies that investigated the influence of *MTHFR* polymorphisms on HD-MTX-related toxicity in patients with NHL reported conflicting results.^3^,^7^,^9^ However, most of these studies included heterogeneous groups of patients, regarding the type of hematologic malignancy and treatment protocols.

We are aware that one of the limitations of our study is the small sample size; however, it was population-based and included a homogenous group of paediatric patients with lymphoblastic T-cell NHL treated according to BFM protocols in Slovenia over the past 17 years. Therefore, the chances of reporting and detection biases due to random sampling were minimized.

In conclusion, our study suggests that *MTHFR* 677C>T polymorphism might modulate MTX pharmacokinetics, and hence influence HD-MTX-related toxicity in patients with lymphoblastic T-cell NHL treated according to BFM protocols.

## Figures and Tables

**TABLE 1.** Clinical and treatment characteristics of paediatric patients with lymphoblastic T-cell non-Hodgkin lymphoma (n = 29)

**Characteristic**	**n (%)**	**Mean (± SD)**
**Median age, years**^a^		11.0 (1.0–18.0)
**Gender**		
Male	25 (86.2)	
Female	4 (13.8)	
**Treatment protocol**		
BFM86	2 (6.9)	
BFM90	9 (31.0)	
BFM95	9 (31.0)	
ICBFM02	9 (31.0)	
**MTX dose, mg/m**^2^		
2000	1 (3.5)	
5000	28 (96.5)	
**Any cycle with delayed MTX clearance**	14 (46.7)	
**Cmax, μmol/l**^b^		49.9 (± 31.2)
AUC1, μmol*h/l^c^		323.3 (± 250.6)
AUC2, μmol*h/l^c^		356.8 (± 215.6)
AUC3, μmol*h/l^d^		297.7 (± 202.0)
AUC4, μmol*h/l^e^		418.4 (± 340.2)
**Treatment outcome**		
First remission	21 (72.4)	
Event^f^	8 (27.6)	

AUC = area under the time-concentration curve after first (1), second (2), third (3), and fourth (4) application; BFM = Berlin-Frankfurt-Münster; Cmax = maximal MTX plasma concentration; ICBFM = intercontinental Berlin-Frankfurt-Münster; MTX = methotrexate; SD = standard deviation

a Age is presented as median (range);

b Data missing for 2 (6.9%) patients;

c Data missing for 8 (27.6%) patients;

d Data missing for 11 (37.9%) patients;

e Data missing for 10 (34.5%) patients;

f Event was defined as disease relapse at any site, death from any cause, or the occurrence of second malignant neoplasm

**TABLE 2.** Prevalence of high-dose methotrexate-related toxicities in the group of paediatric patients with lymphoblastic T-cell non-Hodgkin lymphoma (n = 27)

**Toxicity**	**Number of patients**	**(%)**
anaemia grade ≥ 2	5	(17.2)
leucopoenia grade ≥ 2	6	(20.7)
thrombocytopenia grade ≥ 2	7	(24.1)
hepatotoxicity grade ≥ 1^b^	16	(55.2)
renal toxicity	1	(3.5)
gastrointestinal toxicity	2	(6.9)
mucositis grade ≥ 1	8	(27.6)

b Data on hepatotoxicity missing for 4 (13.8%) patients

**TABLE 3.** The influence of *MTHFD1*, *MTHFR,* and *TYMS* polymorphisms on high-dose methotrexate-related toxicity in pediatric patients with non-Hodgkin lymphoma (n = 28)

**Polymorphism**	**Genotype**	**Number (%)**	**Anaemia grade ≥ 2^a^**		**Leucopoenia grade ≥ 2**		**Thrombocytopenia grade ≥ 2**		**Hepatotoxicity grade ≥ 1^b^**		**Mucositis grade ≥ 1**	

			**OR (95% CI)**	**P**	**OR (95% CI)**	**P**	**OR (95% CI)**	**P**	**OR (95% CI)**	**P**	**OR (95% CI)**	***P***
***MTHFD1* 1958G>A**	GG	11 (37.9)	Reference	0.923	Reference	0.263	Reference	0.592	Reference	0.496	Reference	0.406
	GA	16 (55.2)	0.94		3.75		1.67		0.51		2.18	
	AA	2 (6.9)	(0.13-6.78)		(0.37–37.95)		(0.26–10.79)		(0.08–3.49)		(0.35–13.76)	
***MTHFR* 677C>T**	CC	16 (55.2)	Reference	0.164	Reference	**0.006^c^**	Reference	**0.041**	Reference	0.087	Reference	0.901
	CT	11 (37.9)	5.33		**1.86**		**11.14**		0.13		1.11	
	TT	2 (6.9)	(0.51–56.24)		**(1.12–3.07)**		**(1.11–112.01)**		(0.01–1.34)		(0.21–5.80)	
***MTHFR* 1298A>C**	AA	12 (41.4)	Reference	0.908	Reference	0.158	Reference	0.069	Reference	0.968	Reference	0.824
	AC	15 (51.7)	1.13		0.25		0.17		0.96		1.21	
	CC	2 (6.9)	(0.15–8.21)		(0.04–1.71)		(0.03–1.14)		(0.16–5.80)		(0.22–6.61)	
***TYMS* 2R>3R**	2R/2R	7 (24.1)	Reference	0.700	Reference	0.563	Reference	0.246	Reference	0.858	Reference	0.379
	2R/3R	12 (41.4)	1.60		2.00		0.33		1.20		0.44	
	3R/3R	10 (34.5)	(0.15–17.41)		(0.19–20.90)		(0.05–2.13)		(0.16–8.80)		(0.07–2.71)	
***TYMS* 2R>3RC/3RG**^d^	Low	14 (48.3)	Reference	0.815	Reference	0.262	Reference	0.284	Reference	0.455	Reference	0.284
	High	13 (46.8)	0.82		3.00		0.36		0.50		0.36	
			(0.11–5.99)		(0.44–20.44)		(0.06–2.34)		(0.08–3.08)		(0.06–2.34)	

CI = confidence interval; OR = odds ratio

*TYMS* low expression genotypes: 2RG/2RG, 2RG/3RC, and 3RC/3RC. *TYMS* high expression genotypes: 2RG/3RG, 3RC/3RG, and 3RG/3RG.

ORs, 95% CIs, and *P* values were calculated by univariable logistic regression and the dominant genetic model was used. Bold characters indicate statistically significant results

a Data on anaemia missing for 1 patients (3.9%);

b Data on hepatotoxicity missing for 4 patients (13.8%);

c *P*-value was calculated using Fisher’s exact test;

d Genotyping data missing for 2 patients (6.7%)

## References

[1] Gregoric B, Zadnik V, Jezersek Novakovic B (2012). The diffuse large B-cell lymphoma – where do we stand now in everyday clinical practice. Radiol Oncol.

[2] Reiter A, Schrappe M, Ludwig WD, Tiemann M, Parwaresch R, Zimmermann M (2000). Intensive ALL-type therapy without local radiotherapy provides a 90% event-free survival for children with T-cell lymphoblastic lymphoma: a BFM group report. Blood.

[3] Faganel Kotnik B, Grabnar I, Bohanec Grabar P, Dolzan V, Jazbec J (2011). Association of genetic polymorphism in the folate metabolic pathway with methotrexate pharmacokinetics and toxicity in childhood acute lymphoblastic leukaemia and malignant lymphoma. Eur J Clin Pharmacol.

[4] Erculj N, Kotnik BF, Debeljak M, Jazbec J, Dolzan V (2012). Influence of folate pathway polymorphisms on high-dose methotrexate-related toxicity and survival in childhood acute lymphoblastic leukemia. Leuk Lymphoma.

[5] Jazbec J, Kitanovski L, Aplenc R, Debeljak M, Dolzan V (2005). No evidence of association of methylenetetrahydrofolate reductase polymorphism with occurrence of second neoplasms after treatment of childhood leukemia. Leuk Lymphoma.

[6] Petra BG, Janez J, Vita D (2007). Gene-gene interactions in the folate metabolic pathway influence the risk for acute lymphoblastic leukemia in children. Leuk Lymphoma.

[7] Imanishi H, Okamura N, Yagi M, Noro Y, Moriya Y, Nakamura T (2007). Genetic polymorphisms associated with adverse events and elimination of methotrexate in childhood acute lymphoblastic leukemia and malignant lymphoma. J Hum Genet.

[8] Kantar M, Kosova B, Cetingul N, Gumus S, Toroslu E, Zafer N (2009). Methylenetetrahydrofolate reductase C677T and A1298C gene polymorphisms and therapy-related toxicity in children treated for acute lymphoblastic leukemia and non-Hodgkin lymphoma. Leuk Lymphoma.

[9] Seidemann K, Book M, Zimmermann M, Meyer U, Welte K, Stanulla M (2006). MTHFR 677 (C-->T) polymorphism is not relevant for prognosis or therapy-associated toxicity in pediatric NHL: results from 484 patients of multicenter trial NHL-BFM 95. Ann Hematol.

